# Implications of Polyploidy and Ploidy Alterations in Hepatocytes in Liver Injuries and Cancers

**DOI:** 10.3390/ijms23169409

**Published:** 2022-08-20

**Authors:** Tomonori Matsumoto

**Affiliations:** Department of Molecular Microbiology, Research Institute for Microbial Diseases, Osaka University, Suita 565-0871, Japan; tomomatsumoto@biken.osaka-u.ac.jp

**Keywords:** polyploidy, polyploidization, ploidy reduction, liver regeneration, hepatocellular carcinoma, chromosomal instability, hepatocyte

## Abstract

Polyploidy, a condition in which more than two sets of chromosomes are present in a cell, is a characteristic feature of hepatocytes. A significant number of hepatocytes physiologically undergo polyploidization at a young age. Polyploidization of hepatocytes is enhanced with age and in a diseased liver. It is worth noting that polyploid hepatocytes can proliferate, in marked contrast to other types of polyploid cells, such as megakaryocytes and cardiac myocytes. Polyploid hepatocytes divide to maintain normal liver homeostasis and play a role in the regeneration of the damaged liver. Furthermore, polyploid hepatocytes have been shown to dynamically reduce ploidy during liver regeneration. Although it is still unclear why hepatocytes undergo polyploidization, accumulating evidence has revealed that alterations in the ploidy in hepatocytes are involved in the pathophysiology of liver cirrhosis and carcinogenesis. This review discusses the significance of hepatocyte ploidy in physiological liver function, liver injury, and liver cancer.

## 1. Introduction

Ploidy is the number of complete sets of chromosomes in a genome. Almost all vertebrate animals, including mammals, are diploid organisms whose somatic cells contain two sets of haploid genomes originally derived from each parent. However, a significant number of hepatocytes in the liver are physiologically polyploid, containing double, quadruple, or more chromosomes. Polyploidy of hepatocytes in the mammalian liver was originally suggested more than a century ago by the observation that the nuclear sizes of hepatocytes in adult rodent livers markedly increased compared with those in young livers [1]. In addition, hepatocytes frequently contain more than one nucleus [2]. These histological findings and a clear correlation between nuclear size and ploidy that was demonstrated later clarified polyploidization of hepatocytes during liver maturation [1]. Furthermore, hepatocyte ploidy is known to increase during aging or liver damage. Although the mechanism underlying the regulation of the ploidy of hepatocytes and its effects on the pathophysiology of the liver are unclear, accumulating evidence indicates that polyploidy and alterations in the ploidy in hepatocytes influence liver homeostasis and diseases both positively and negatively. Whereas some reviews have outlined the advantages and disadvantages of polyploidy in health and disease in various organs [3,4], few papers have highlighted updated discussions about hepatocyte ploidy, especially ploidy alterations, and liver diseases [5,6,7]. This review discusses the significance of hepatocyte ploidy, with respect to physiological liver function, liver injury, and cancer. To obtain a balanced overview of the latest progress on this area, recent articles retrieved from PubMed searches using the keywords hepatocyte and ploidy were critically reviewed and selected based on the relevance to the current topic. This review will give the latest overview and some future perspectives about the implications of polyploidy and ploidy alterations in hepatocytes in liver diseases.

## 2. Physiological Polyploidization of Hepatocytes

### 2.1. Overview of Physiological Polyploidization of Hepatocytes

**Figure 1:**
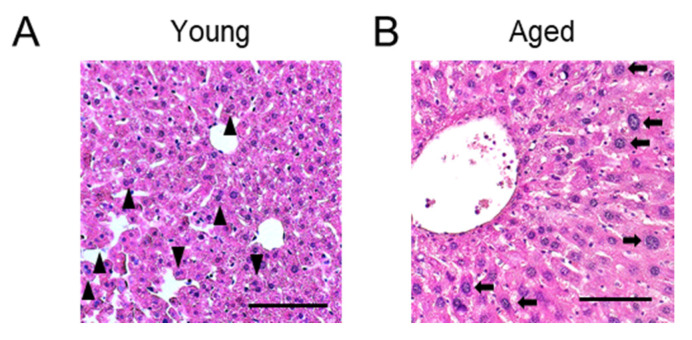
Histological images of livers in young (**A**) and aged (**B**) mice. Representative hematoxylin and eosin staining images are shown. (**A**) The liver of a 6-week-old mouse. Some binucleated polyploid hepatocytes are indicated by arrow heads. (**B**) The liver of a 69-week-old mouse. Polyploid hepatocytes with a large nucleus are indicated by arrows. Scale bars, 100 μm.

Physiological polyploidization of hepatocytes is widely observed in the liver of various mammalian species, including humans and rodents (Figure 1A) [1,8]. Although the frequency of hepatocyte polyploidization differs depending on the species, physiological polyploidization is primarily observed during the course of liver maturation [1,8]. While almost all hepatocytes are diploid in neonatal mice and rats, polyploidization starts immediately prior to weaning in mice and at the time of weaning in rats [9,10,11]. Most (~90%) hepatocytes in the adult rodent liver are polyploid [12]. One of the triggers that induce drastic hepatocyte polyploidization in rodents is the transition in nutritional conditions associated with weaning, as discussed in the subsequent section [10]. In contrast, the increase in physiological polyploidization in humans is slower than that in rodents. Approximately 95% of hepatocytes are diploid in newborns, and polyploid hepatocytes begin to appear in young children [13]. The proportion of polyploid hepatocytes gradually increases with age and reaches up to 20–50% at an age of approximately 40–50 years [13,14,15]. The ploidy levels of hepatocytes that increase during normal development are further enhanced with aging in both rodents and humans (Figure 1B) [13,16]. Furthermore, intensified polyploidization in aged humans has been reported previously [13].

### 2.2. Mechanisms of Polyploidization

The mechanisms of polyploidization can be broadly classified into two types: abnormal cell cycle processes and cell fusion. In the former, genome duplication during the DNA synthesis (S) phase and subsequent abnormal cell cycle progression results in the formation of mononucleated or binucleated polyploid cells. In contrast, cell fusion can be induced in a cell cycle-independent manner and generates polyploid cells with two or more nuclei. Multinucleated skeletal muscle cells are formed by the fusion of mononucleated myoblasts during development [17]. Syncytiotrophoblasts in placental villi are another example of polyploid cells produced by the cell fusion [18].

**Figure 2:**
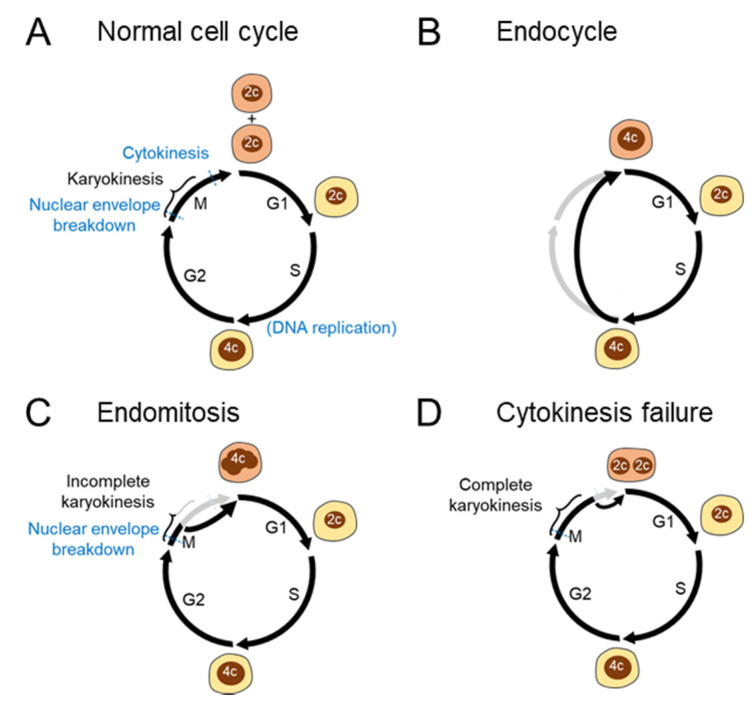
Cell cycle aberrations leading to polyploidization (**A**) Normal cell cycle. A diploid cell gives rise to two diploid daughter cells through mitosis. (**B**) Endocycle. The S and G1 phases are repeated without entering the M phase, leading to the emergence of mononuclear polyploid cells. (**C**) Endomitosis. Although cells enter the M phase with chromosome condensation and disruption of the nuclear envelope, the mitotic process is aborted, leading to the genesis of mononucleated polyploid cells. (**D**) Cytokinesis failure. Cells complete karyokinesis but not the following cytokinesis, generating binucleated polyploid cells. 2c and 4c denote diploid and tetraploid DNA contents, respectively.

Polyploidization resulting from abnormal cell cycle processes can be classified in more detail (Figure 2A–D). The endocycle is a variant cell cycle in which rounds of the S and gap (G) phases are repeated without entering the mitosis (M) phase (Figure 2B). Owing to the lack of mitosis, endocyclic cells become polyploid without exhibiting the features of mitosis, including chromosome condensation and disruption of the nuclear envelope [19]. Endocycles are relatively limited in mammals. Trophoblast giant cells are the best-studied cell types that undergo endocycles in mammals [20]. Tubular epithelial cells in the kidney also undergo endocycles in response to acute kidney injury [21].

In contrast to endocycles, endomitosis is a polyploidization process that is accompanied by mitosis (Figure 2C). Megakaryocytes are a well-known polyploid cell type in mammals, and their polyploidization results from the abortion of mitosis during late anaphase [22]. As chromosomes segregate incompletely during each M phase in polyploidizing megakaryocytes, polyploid megakaryocytes exhibit a polylobulated mononucleus [22]. While such an endomitosis process without complete karyokinesis leads to the formation of mononucleated polyploid cells, failed cytokinesis after complete karyokinesis results in the formation of binucleated polyploid cells (Figure 2D). Such an incomplete cytokinesis process is sometimes included as an extended definition of endomitosis. Cytokinesis failure is the primary mechanism underlying physiological polyploidization in hepatocytes and cardiomyocytes [23]. Podocytes in the kidney are also unable to complete cytokinesis [4], and multinucleated cells are physiologically observed in the kidney [24]. Under diseased conditions, hepatocytes can become polyploid in other ways, including mitotic slippage and cell fusion, which will be discussed later.

### 2.3. Molecular Mechanisms Underlying Physiological Polyploidization in Hepatocytes

The mechanism underlying the regulation of physiological polyploidization has not been completely elucidated. However, some key regulators have been demonstrated in rodent studies. The Desdouets group elegantly showed that after weaning, impaired organization of the actin cytoskeleton during late anaphase leads to the failure of RhoA activation required for cytokinesis in rat hepatocytes [25]. In addition, insulin signaling, which can be enhanced by the suckling-to-weaning transition, induces cytokinesis failure in hepatocytes via activation of the downstream PI3K/Akt pathway in rats and mice [10]. MicroRNAs, especially miR-122, are also important for inducing hepatocyte polyploidization [11]. miR-122 is the predominant miRNA found in hepatocytes, and liver-specific miR-122 knockout reduces the number of binucleated hepatocytes by 60–70% in mice [11]. Importantly, miR-122 is the most highly expressed miRNA in hepatocytes in 2–3-week-old mice when physiological binucleation of mouse hepatocytes is initiated, and miR-122 suppresses the expression of some cytokinesis-associated genes, including RhoA [11]. Other genetically engineered mouse strains have also been reported to exhibit altered hepatocyte ploidy. Physiological polyploidization of hepatocytes is impaired in mice whose livers are deficient in atypical E2Fs, E2f7, and E2f8 [26]. Between the two atypical E2F proteins, E2F8 seems to play a more important role in inducing hepatocyte polyploidization than E2F7, and E2f8-knockout and E2f7/E2f8 double-knockout livers predominantly comprise diploid hepatocytes [26]. E2F8 is supposed to antagonize transcriptional activation by E2F1, and genes whose transcription is commonly targeted by both E2F1 and E2F8 include regulators of cytokinesis, such as Ect2, Mklp1, and Racgap [26]. Genes associated with the circadian period are also involved in the physiological hepatocyte polyploidization [27]. Hepatocyte polyploidization is markedly accelerated around the central vein in Per-null mice deficient in the three Period genes, namely, Per1, Per2, and Per3 [27]. Mechanistically, Mkp1-mediated circadian modulation of the Erk1/2 activity is impaired in Per-null mice, which enhances the polyploidization [27]. Moreover, other models in which regulators of cell cycles, such as RB, are manipulated also show altered ploidy status in hepatocytes [28,29]. Although it is still unclear whether these molecules play critical roles in the polyploidization of human hepatocytes, these findings provide a strong foundation for elucidating the detailed mechanisms underlying ploidy regulation in human hepatocytes.

### 2.4. Significance of Physiological Polyploidization

As physiological polyploidization of hepatocytes is a universal phenomenon in humans and rodents, physiologically polyploid hepatocytes would have some advantages in the development or maintenance of liver function. Typically, polyploidization is considered to induce terminal cellular differentiation. Physiological polyploidization in cardiomyocytes and megakaryocytes is accompanied by their differentiation [30]. Hence, the physiological polyploidization observed during liver maturation may contribute to the enhancement of liver function.

To examine functional differences between diploid and polyploid hepatocytes, some studies have analyzed differentially expressed genes between them. However, gene expression seems to be almost unaffected by hepatocyte ploidy. Microarray and RNA sequencing analyses of sorted diploid and polyploid mouse hepatocytes did not identify clear transcriptional differences between diploid and polyploid hepatocytes [31,32]. On the other hand, some studies have suggested that polyploidy in hepatocytes affects cellular biological processes, such as lipid and carbohydrate metabolism; however, no consensus has been established with respect to the results [33,34,35]. A recent study in which mouse liver cells were analyzed by single-nucleus RNA sequencing after ploidy-based sorting provided many important insights into the expression patterns of polyploid hepatocytes [36]. In this study, Richter et al. showed that the transcriptomes of diploid and polyploid hepatocytes are globally very similar; however, some genes related to metabolism are differentially expressed between these two groups [36]. Importantly, the expression of such ploidy-related genes is conditioned by their position within the hepatic lobule [36]. In other words, polyploid hepatocytes are preferentially located in the pericentral zone, as previously suggested [37,38], and hepatic metabolic zone is the major determinant of gene expression levels in polyploid hepatocytes [36]. In addition, impairment of hepatocyte polyploidization in E2f7/E2f8 double-knockout mice has no impact on liver differentiation, zonation, or metabolism [26]. These findings challenge the traditional hypothesis that polyploidization promotes terminal differentiation in hepatocytes and is beneficial for adult liver function, including metabolism. The significance of polyploidy in liver function requires further study under both healthy conditions and those involving stress.

Another advantage of polyploidy is its genomic redundancy. One of the important functions of the liver is detoxification, and hepatocytes are constantly exposed to genotoxins, such as aldehydes generated during alcohol metabolism. Polyploidy is supposed to act as a “buffer” against genotoxic damage, particularly that attributed to the loss-of-function genetic alterations, because of redundant genetic alleles [39]. This advantage can be associated with protection from carcinogenesis and will be discussed in more detail later. In addition, hepatocytes exhibit bursty gene transcription and extensive variability in gene expression, and polyploidy may contribute to the reduction in the noise in gene expression caused by transcription bursting [40]. Lower transcriptional variability in polyploid hepatocytes than in diploid cells has also been observed using single-nucleus RNA sequencing analysis [36]. As the liver is exposed to multiple bioactive substances derived from the intestine, as well as genotoxins, polyploidy may also contribute to the mitigation of unexpected gene expression alterations triggered by external factors.

Taken together, physiological polyploidization in hepatocytes may have some advantages, with respect to multiple and wide range of liver functions, such as metabolism, but this has not been confirmed. In contrast, genomic redundancy of polyploidy may contribute to the mitigation of unfavorable damage or stimulation in hepatocytes, which is likely to be observed in the liver, as it is the main organ of detoxification and the first organ to filter the portal circulation.

## 3. Polyploid Hepatocytes in Acute and Chronic Liver Damage

### 3.1. Ploidy Alterations in Liver Damage

**Figure 3:**
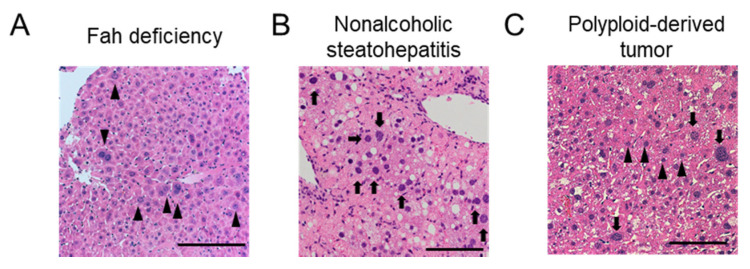
Ploidy alterations in mouse livers with various liver diseases. Representative hematoxylin and eosin staining images are shown. (**A**) Fah deficiency, a model of familial tyrosinemia [37]. Some mononucleated and binucleated polyploid hepatocytes are indicated by arrow heads. (**B**) Nonalcoholic steatohepatitis [46]. Some polyploid hepatocytes with a large nucleus are indicated by arrows. (**C**) Tumor derived from polyploid hepatocytes [46]. Please note that cancer cells derived from polyploid hepatocytes frequently undergo ploidy reduction and that the tumor is composed of both cancer cells with a small nucleus (arrow heads) and those with a large nucleus (arrows). Scale bars, 100 μm.

Various types of liver injuries induce ploidy alterations in hepatocytes in the adult liver after physiological polyploidization. Enhancement of polyploidization has been observed in human chronic viral hepatitis [15], as well as in nonalcoholic fatty liver disease [41]. Some rodent models of liver injuries, such as those attributed to iron accumulation [42], copper accumulation [43], tyrosinemia [44], and partial hepatectomy [45], have also demonstrated enhanced hepatocyte polyploidization (Figure 3A,B). Although it is difficult to discriminate between physiological polyploidization and a pathological one that may be induced by aging-related hepatocyte changes such as accumulation of DNA damages and dysfunctional mitochondria, polyploid hepatocytes also accumulate in aged livers [6,9].

Whereas physiological polyploidization of hepatocytes is predominantly induced by incomplete cytokinesis, polyploidization under pathological conditions can be attributed to various mechanisms. Gentric et al. showed that in livers with nonalcoholic fatty liver disease, DNA damage induced by oxidative stress leads to the inhibition of CDK1 activation and induces G2/M arrest via activation of the ATR/p53/p21 pathway, which results in polyploidization [41]. A similar successive process that associates DNA damage and polyploidization has been reported during the induction of senescence [47]. Various types of liver damage that accompany DNA damage and/or senescence induction in hepatocytes [48] would lead to polyploidization of hepatocytes via arrest in the G2/M phase mediated by p53 activation. Mitotic slippage observed after prolonged spindle assembly checkpoint (SAC) activation is another type of abnormal cell cycle progression that leads to polyploidization [49]. SAC ensures accurate attachment between chromosomes and spindle microtubules, and its activation prevents the progression from metaphase to anaphase. Inextricable SAC activation induces mitotic cell death but is sometimes bypassed, leading to premature exit from mitosis, which is called mitotic slippage [49]. Mitotic slippage is particularly observed in cancer cells treated with microtubule-affecting drugs, and it generates mononucleated polyploid cells [49]. In addition, the hepatitis B virus (HBV) X protein is reported to bind to BUB1B, a component of the mitotic checkpoint complex, and enhance the mitotic slippage [50]. HBV X protein also induces abortive mitosis and abnormal polyploidization by activating p38 and upregulating the expression of PLK1 [51]. Consistently, human hepatocytes infected with HBV show an enhanced polyploidization [51]. Moreover, cell fusion can be observed in an injured liver. Cell fusion between hepatocytes and bone marrow-derived macrophages is observed when injured livers of fumarylacetoacetate hydrolase (Fah)-deficient mice, a model of familial tyrosinemia type I, are regenerated by the transplantation of bone marrow cells [52,53].

Interestingly, both polyploidization and reduction of ploidy occur in injured livers. The generation of diploid hepatocytes from polyploid cells was first demonstrated in the fusion-derived hepatocytes mentioned above [54]. Moreover, the transplantation of sorted polyploid hepatocytes into the recipient livers resulted in the emergence of diploid cells during the proliferation of donor polyploid cells [55]. Such ploidy reduction (depolyploidization) occurs via multipolar mitosis [55] and is detected during various chronic liver injuries [37]. Intriguingly, depolyploidized diploid cells readily undergo re-polyploidization in subsequent cell cycles [37,55]. Thus, ploidies of hepatocytes can be altered dynamically during liver injury, and polyploidization and depolyploidization are induced by various mechanisms under pathological conditions.

### 3.2. Liver Regeneration by Polyploid Hepatocytes

As described previously, polyploidization is considered to be associated with terminal cell differentiation, and the proliferation of terminally differentiated polyploid cells is arrested in many cell types [19,30]. In some organ systems, development and regeneration stem exclusively from diploid cells, whereas polyploid cells function as differentiated cells and do not proliferate for regeneration. For example, hematopoietic stem/progenitor cells are diploid, and differentiated polyploid megakaryocytes do not proliferate [22]. Heart regeneration is also attributed to diploid cardiomyocytes, although there is lack of clarity regarding whether polyploid cardiomyocytes are completely unable to proliferate [56,57]. These observations, as well as the fact that polyploidization frequently induces cell cycle arrest in nontransformed cells in vitro [58], led to the hypothesis of the “tetraploidy checkpoint” in the past [59].

However, hepatocytes do not seem to have such a definitive “polyploidy checkpoint,” and they retain their proliferative capacities even after polyploidization. The liver has an extensive regenerative capacity, and the proliferation of hepatocytes primarily leads to the regeneration of the liver damaged by surgical resection or various liver injuries. After 70% partial hepatectomy in mice, during which the most dynamic liver regeneration is observed, almost all hepatocytes, including polyploid cells, enter cell cycle and undergo DNA synthesis in the S phase [60]. Approximately half of these hepatocytes do not enter the M phase, resulting in polyploidization and hepatocyte hypertrophy, whereas others complete mitosis and proliferate [60]. Polyploid hepatocytes proliferate in this way after massive surgical resection of the liver, although the initiation of the proliferation of polyploid hepatocytes for regeneration after hepatectomy is reported to be relatively delayed compared to that of diploid hepatocytes [61].

In contrast to such transient liver regeneration, whether polyploid hepatocytes continuously proliferate in chronic liver injuries remained unclear because it was difficult to distinguish between the emergence of polyploid cells by polyploidization of diploid cells and polyploid cell proliferation. A novel system to trace polyploidy using multicolor reporter mice was used to overcome this challenge, and the results confirmed the persistent proliferation of polyploid hepatocytes in chronically damaged livers [37]. In mice heterozygous for a multicolor reporter allele, polyploid cells are labeled by co-expression of multiple colors, and the proliferation of polyploid cells can be traced [37]. This system clearly demonstrated that the majority of polyploid hepatocytes, even octaploid hepatocytes, proliferate continuously during various types of chronic liver injury [37]. Notably, proliferating multicolored polyploid hepatocytes sometimes give rise to monocolored daughter cells, indicating ploidy reduction during regeneration driven by polyploid cells in chronically injured livers [37]. Moreover, polyploid hepatocytes repeatedly divide to maintain normal hepatocyte turnover during aging [16]. Taken together, these findings indicate that abundant polyploid hepatocytes have extensive regenerative potential and participate in liver regeneration.

### 3.3. Significance of Ploidy Alterations in Liver Injuries

As discussed before, the genomic redundancy of polyploidy would have advantages in mitigating genotoxic damage and/or unfavorable gene expression noise, especially in liver injuries. In addition, polyploidization and ploidy reduction have been reported to enhance genetic diversity in the liver [6]. Duncan et al. elegantly showed that aneuploidy and genetic variation induced by ploidy alterations enhance the adaptation of hepatocytes to chronic liver injury [44,55,62]. They utilized Fah-deficient mice (Fah−/−) with tyrosinemia-related liver injury and mice heterozygous for a mutation in the homogentisate 1,2-dioxygenase (Hgd) gene (Hgd+/−). Lethal hepatocyte damage was induced in Fah−/− mice shortly after the withdrawal of 2-(2-nitro-4-trifluoro-methyl-benzoyl)-1,3-cyclo-hexanedione (NTBC) treatment, which prevented the accumulation of toxic intermediates during tyrosine catabolism [62]. In contrast, in Fah−/−Hgd+/− mice, the liver demonstrated extensive damage after the discontinuation of NTBC treatment but was subsequently regenerated by healthy hepatocytic nodules [62]. Hgd is an enzyme that acts upstream of Fah, and homozygous loss of the Hgd gene can prevent hepatocyte damage caused by Fah deficiency [63]. Consistently, healthy hepatocytes that mediate repopulation in Fah−/−Hgd+/− mice lack a functional Hgd due to acquisition of point mutations in the wild-type Hgd gene (~25%) or the loss of chromosome 16 encoding the Hgd gene [62]. These results clearly indicate that a specific aneuploid karyotype can result in the adaptation of hepatocytes to chronic liver injury and is selected during the liver regeneration [62]. Such a numerical chromosome imbalance that can lead to genetic variation is enhanced by ploidy reduction via multipolar mitosis during liver regeneration [46,55]. Furthermore, in a comparison between the diploid-dominant livers of E2f7/E2f8 double-knockout mice and polyploid-dominant livers of control mice, diploid-dominant livers harbored fewer aneuploidies in hepatocytes and were more susceptible to tyrosinemia-induced liver injury than polyploid-dominant livers, suggesting that aneuploidy mediated by polyploid cells enhances the adaptation of hepatocytes to liver injuries [44].

The frequencies of aneuploidy in the liver reported in the literature vary depending on the experimental approach. However, single-cell whole-genome sequencing has detected aneuploidy in a certain percentage of human and mouse hepatocytes, especially in polyploid cells [64,65]. Although the frequency of aneuploidy would be relatively low (<5%), there should be a significant number of aneuploid hepatocytes in the body, given the number of hepatocytes in the entire liver [6]. Further studies are needed to confirm whether aneuploidy that is likely to occur during the course of polyploidization, proliferation, and ploidy reduction in hepatocytes enhances genetic variation in hepatocytes, promoting hepatocyte adaptation to various types of liver injuries. Similar adaptive evolution by aneuploidy has been reported in yeast [66] and is also involved in the evolution of cancer cells, as discussed later.

## 4. Polyploidy and Ploidy Alterations in the Development of Liver Cancer

### 4.1. Correlation between Polyploidization and Carcinogenesis

The polyploidization process, including cytokinesis failure, mitotic slippage, and cell fusion, accompanies centrosome amplification [67,68]. Over a century ago, Boveri et al. suggested that supernumerary centrosomes lead to chromosome missegregation and aneuploidy, resulting in cancer development [69]. The phenomenon that leads to the development of aneuploidy is called chromosomal instability (CIN) [70], and centrosome amplification indeed leads to CIN and cancer development in mice [71]. Thus, proliferating polyploid cells with supernumerary centrosomes are likely to accompany chromosome missegregation and may lead to carcinogenesis.

Various lines of evidence have suggested that polyploidization can promote tumorigenesis, probably by enhancing CIN [72]. The first direct evidence of polyploidy-driven tumorigenesis was obtained with the elucidation of the fact that polyploid p53-null mammary cells are more prone to tumorigenesis via chromosome missegregation during mitosis than their diploid counterparts [73]. Studies examining human specimens have also demonstrated implications of polyploidization in cancer initiation. The first well-elucidated example of polyploidy-driven cancer initiation is Barrett esophagus carcinogenesis; polyploid populations in premalignant Barrett esophagus are predisposed to aneuploidy and carcinogenesis [74]. A recent study of human pan-cancer genomes that analyzed the evolutionary history of various cancers also estimated that polyploidization generally occurs after some early key events, such as TP53 mutations, and leads to the accumulation of copy number alterations, resulting in the emergence of the most recent common ancestor populations [75].

### 4.2. Impacts of Hepatocyte Ploidy on Liver Cancer Development in Rodent Studies

Despite the aforementioned results suggesting the implications of polyploidization in the initiation of various cancers, it is still controversial whether polyploidy in hepatocytes promotes or suppresses the development of hepatocellular carcinoma (HCC). One important aspect of polyploidy is that it may protect cells from malignant transformation by buffering genotoxic damage and reducing the chance of tumor suppressor loss. As the liver physiologically contains abundant polyploid hepatocytes, unlike other organs, the influence of hepatocyte ploidy on carcinogenesis has been studied directly using rodent models with unique ploidy characteristics. Some mouse models with increased or decreased numbers of polyploid hepatocytes in their livers demonstrated that livers with enhanced polyploidy have a lower risk of cancer development compared to their counterparts [32,39,61,76]. These results suggest a cancer-preventive effect of hepatocyte polyploidy, although it is unclear whether this effect can be attributed to the redundancy of the polyploid genome that protects from the loss of heterozygosity of tumor suppressor genes or from differences in the cellular proliferative potential [61].

However, polyploid hepatocytes are not completely protected from carcinogenesis (Figure 3C). Lineage-tracing analysis using multicolor reporter mice that can visualize the proliferation of polyploid cells demonstrated that physiologically polyploid hepatocytes readily serve as a source of cancer [46]. More importantly, the ploidy reduction (depolyploidization) that can occur during liver regeneration negates the protective effect of polyploidy in preventing tumor suppressor loss [46]. In fact, in multicolor tracing mice, the majority of tumors derived from bicolored polyploid cells demonstrated a loss of expression of either one or both reporters in the entire tumor, suggesting frequent ploidy reduction, especially in the early phase of tumorigenesis derived from polyploid hepatocytes [46]. Enhancement of carcinogenesis by ploidy reduction was elucidated by competitive assays, particularly in carcinogenesis driven by tumor suppressor gene loss [46]. The implications of ploidy reduction in the initiation of HCC have also been demonstrated in another independent study [77]. In a detailed analysis of diethylnitrosamine (DEN)-induced hepatocarcinogenesis in mice by Lin et al., DEN treatment induced DNA damage and hyperpolyploidization in hepatocytes around the centrilobular region in the mouse liver, and centrilobular hepatocytes preferentially gave rise to preneoplastic lesions [77]. Intriguingly, the nuclear and cellular sizes of cells in the preneoplastic lesions were smaller than those of the surrounding polyploidized cells, suggesting ploidy reduction during their transformation into preneoplastic cells [77]. The involvement of polyploidization and subsequent ploidy reduction in hepatocarcinogenesis was further suggested by the modulation of aurora kinase B (Aurkb), which plays a role in hepatocyte polyploidization and liver cancer development [77].

### 4.3. Consideration of Carcinogenic Risks Related to Hepatocyte Ploidy

As discussed above, physiological polyploidization seems to protect cells from carcinogenesis. On the other hand, polyploid hepatocytes readily give rise to cancers in some models, and polyploidization is considered to be an important driver of carcinogenesis in many cancers. Certain perspectives related to the dynamics of polyploid hepatocytes may explain the seemingly contradictory results, with respect to the correlation between hepatocyte ploidy and liver cancer development. Although polyploidy in itself can act as buffer against genotoxic damage and oncogenic genomic alterations, the proliferation of polyploid hepatocytes, especially in chronically damaged livers, would lead to chromosome missegregation. Mitosis of polyploid cells leads to CIN [78,79], although the frequency of chromosome missegregation in proliferating polyploid hepatocytes in vivo has not been determined. Furthermore, ploidy reduction, which can be induced by complex multipolar mitosis, is suggested to enhance CIN [46,55]. Thus, mitosis and ploidy reduction after polyploidization would play critical roles in polyploidy-driven carcinogenesis.

**Figure 4:**
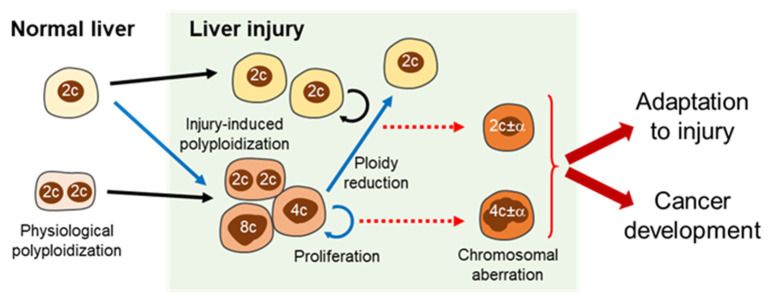
Significance of ploidy alterations in liver injuries and cancer development. Hepatocyte polyploidization is enhanced in injured livers. Both diploid and polyploid hepatocytes proliferate for regeneration in injured livers, and ploidy reduction is sometimes induced during regeneration driven by polyploid hepatocytes. These processes (blue arrows) are likely to be a source of chromosome aberrations in hepatocytes (red dashed arrows). Moreover, the chromosome aberrations enhance genetic variation in hepatocytes, increasing the chance of hepatocyte adaptation to liver injuries as well as liver cancer initiation. 2c, 4c, and 8c denote diploid, tetraploid, and octaploid DNA contents, respectively. 2c ± α and 4c ± α indicate aneuploidy.

Given that proliferating polyploid hepatocytes are a high-risk factor for the development of carcinogenesis, even if the risk is mitigated by polyploid redundant genomes, regeneration of chronic liver injuries by the continuous proliferation of polyploid hepatocytes may be unsafe. Intriguingly, however, ploidy reduction and carcinogenesis were suppressed in polyploid hepatocytes after prolonged proliferation [46]. This implies that polyploid hepatocytes become chromosomally stable during multiple cycles of mitosis; however, the mechanisms remain unknown. In other words, the process of polyploidization and subsequent chromosomally unstable mitosis, such as multipolar mitosis leading to ploidy reduction (i.e., fluctuations in hepatocyte ploidy) may drive carcinogenesis (Figure 4). Further studies are required to elucidate the significance of ploidy alterations in carcinogenesis, for example, to determine whether unprogrammed polyploidization induced by hepatocyte damage has a unique impact on CIN and hepatocarcinogenesis, which is different from physiological polyploidization.

### 4.4. Polyploidy in Hepatocellular Carcinoma

Polyploidization, which is often referred to as whole-genome doubling in the field of cancer genome research, is also frequently observed in cancer cells after malignant transformation. Whole-genome doubling has been recurrently detected in various types of human solid cancers [80,81], and a recent large-scale analysis of sequencing data of human cancer genomes showed that approximately 36% of tumors demonstrated at least one whole-genome doubling event during their evolution [82]. In hepatobiliary cancers, approximately 34% and 22% of HCCs and cholangiocarcinomas, respectively, are supposed to have undergone whole-genome doublings [82]. While some of these genome-duplication events detected in primary tumors may have occurred early in tumorigenesis and driven cancer initiation, others may have also taken place during the advanced stage of cancer progression. Whole-genome doublings are more frequently observed in metastatic cancers than in primary tumors in some types of cancers, suggesting the role of polyploidization in metastasis [83]. Whole-genome doublings have also been implicated in the acquisition of drug resistance in cancer cells [84]. As accumulating evidence indicates that genome-duplication events and proliferation of polyploid cancer cells leads to CIN and cancer progression, treatments specifically targeting the proliferation of polyploid cancer cells can be a promising new strategy for cancer treatment. To date, there is no established treatment for polyploid cancer cells, but recent studies elucidated some candidate targets such as KIF18A and IL-6 [82,85,86].

In contrast to such pan-cancer studies analyzing cancer genomic data, detailed clinicopathological analyses focusing on HCC and the ploidies of cancer cells are quite limited. Some studies have analyzed DNA ploidies of human HCC using flow cytometry and detected diploid and “aneuploid” HCCs [87,88,89,90]. However, polyploidy has rarely been reported, perhaps because some polyploid HCCs were sometimes included in “aneuploid” HCCs. In addition, the characteristics of diploid HCCs, especially in terms of tumor aggressiveness, are inconsistent between these studies [87,88,89]. Such uncertain results may be attributed to the difficulty in flow cytometric analysis. Inadequate dissociation of tumor tissues can easily lead to distorted histograms of DNA ploidies, and various non-tumor cells, most of which are presumably diploid, are inevitably included in the results. A study by Bou-Nader et al. recently evaluated nuclear ploidies in HCC sections using an imaging cytometry technique [91]. They demonstrated that nuclear ploidies of HCC correlate with histological and molecular features, such as the overexpression of proliferation markers, and that highly polyploid tumors are associated with a poor prognosis [91]. Further studies are needed to confirm the characteristics of polyploid HCCs and elucidate the involvement of ploidies in the pathophysiology of HCCs.

## 5. Conclusions

Alterations in hepatocyte ploidy are considered to be involved in the pathophysiology of the normal liver, various types of liver injuries, and liver cancer evolution. Polyploidization and ploidy reduction appear to have both advantages and disadvantages. For example, increased incidence of aneuploidy (i.e., CIN) during ploidy alterations may enhance the adaptation of hepatocytes to liver injuries, as well as increase the carcinogenic risk, thereby acting as a double-edged sword. In addition, polyploidization in many cell types leads to arrested proliferation, which may protect against carcinogenesis. However, hepatocytes apparently undergo proliferation after polyploidization. Other than cancer cells, only a few types of nontransformed cells such as hepatocytes and mouse embryonic stem cells [92] have been reported to proliferate and undergo ploidy reduction after polyploidization. Given that polyploid hepatocytes seem to become chromosomally stable during multiple cycles of mitosis [46], hepatocytes may have a special property to suppress CIN while permitting ploidy alterations. Future studies elucidating the mechanisms by which hepatocytes regulate mitosis after polyploidization and manage chromosome segregation with an appropriate frequency of errors will open the door to the development of novel treatments focusing on liver regeneration and cancer prevention. In particular, drugs to prevent ploidy reduction and enhance chromosomal stability would be a novel approach to suppress hepatocarginogenesis as well as cancer progression in various organs.

## Data Availability

Not applicable.
